# Interplant Aboveground Signaling Prompts Upregulation of Auxin Promoter and Malate Transporter as Part of Defensive Response in the Neighboring Plants

**DOI:** 10.3389/fpls.2017.00595

**Published:** 2017-04-19

**Authors:** Connor Sweeney, Venkatachalam Lakshmanan, Harsh P. Bais

**Affiliations:** ^1^Delaware Biotechnology Institute, NewarkDE, USA; ^2^Department of Plant and Soil Sciences, University of Delaware, NewarkDE, USA; ^3^Wilmington Charter School, WilmingtonDE, USA

**Keywords:** beneficial microbes, *Bacillus subtilis*, malic acid, microbiome, VOCs, wounding

## Abstract

When disrupted by stimuli such as herbivory, pathogenic infection, or mechanical wounding, plants secrete signals such as root exudates and volatile organic compounds (VOCs). The emission of VOCs induces a response in the neighboring plant communities and can improve plant fitness by alerting nearby plants of an impending threat and prompting them to alter their physiology for defensive purposes. In this study, we investigated the role of plant-derived signals, released as a result of mechanical wounding, that may play a role in intraspecific communication between *Arabidopsis thaliana* communities. Plant-derived signals released by the wounded plant resulted in more elaborate root development in the neighboring, unwounded plants. Such plant-derived signals also upregulated the Aluminum-activated malate transporter (*ALMT1*) responsible for the secretion of malic acid (MA) and the DR5 promoter, an auxin responsive promoter concentrated in root apex of the neighboring plants. We speculate that plant-derived signal-induced upregulation of root-specific *ALMT1* in the undamaged neighboring plants sharing the environment with stressed plants may associate more with the benign microbes belowground. We also observed increased association of beneficial bacterium *Bacillus subtilis* UD1022 on roots of the neighboring plants sharing environment with the damaged plants. Wounding-induced plant-derived signals therefore induce defense mechanisms in the undamaged, local plants, eliciting a two-pronged preemptive response of more rapid root growth and up-regulation of *ALMT1*, resulting in increased association with beneficial microbiome.

## Introduction

Studies have also shown that aboveground pathogen and herbivore attack shifts microbiome activity at the belowground level (Yang et al., 2011; Song et al., 2016). We have shown previously that plants under attack by pathogenic bacteria induce a shoot-to-root systemic signal, inducing roots to recruit benign, protective microbes (Rudrappa et al., 2008; Lakshmanan et al., 2012). The shoot-to-root systemic signal triggers a malate transporter (*ALMT1*), which has been shown to also be activated in response to other abiotic responses (Kochian, 1995; Kobayashi et al., 2007). The *ALMT1* transporter prompts the secretion of tricarboxylic acid cycle intermediate L-malic acid (MA) from *Arabidopsis thaliana* roots, which augments recruitment of the beneficial rhizobacterium *Bacillus subtilis* UD1022-a plant–microbial interaction that decreases susceptibility to many foliar pathogens (Rudrappa et al., 2008; Kumar et al., 2012; Lakshmanan et al., 2012, 2014; Lakshmanan and Bais, 2013). Like most Gram-positive bacteria, *B. subtilis* creates an extracellular matrix composed mainly of proteins and exopolysaccharides (Marvasi et al., 2010). It is documented that the ability of *B. subtilis* to colonize plant roots via biofilm formation is an important feature that adds to the plant growth promotion and biocontrol activity (Lakshmanan et al., 2014; Allard-Massicotte et al., 2016). When colonized on plant roots, *B. subtilis* forms a sort of protective armor around its host by secreting antimicrobial compounds, namely the lipopeptide surfactin, that inhibit the growth of fungi, nematodes, and pathogenic bacteria like *Pseudomonas syringae* (Vlamakis et al., 2013). It is also known that both biotic and abiotic stress may modulate the root microbiome (Erlacher et al., 2015; Lakshmanan, 2015). In addition to root-exuded chemicals, plants are known to signal other plants, microbes, nematodes, and insects via emission of non-polar volatile organic compounds (VOCs) (Delory et al., 2016). The root secretions and VOCs serve as a plant’s arsenal of chemical signals that induce change in inter/intraplant interactions (Baldwin et al., 2006; Owen et al., 2007; Delory et al., 2016). It is known that plant-derived chemical compounds impact plants response against microbes and also mediate changes in plant development via upregulation of growth regulator response (Dudareva et al., 2013).

One important plant growth hormone is the indole-3-acetic acid, a natural auxin, which is responsible for plant cell division and elongation and serves as a signaling molecule in the process of organ and root offshoot initiation (Vanneste and Friml, 2009). The role of auxin in mitigating plant stress has also been noted, specifically to inhibit photo-respiratory-dependent cell death in *Arabidopsis thaliana* (Kerchev et al., 2015). Root growth and differentiation is important for plant survival and its adaption to the extreme environment (Villordon et al., 2014). It is known that root branching and architecture is mediated by both biotic and abiotic factors (Villordon et al., 2014; Khan et al., 2016). Endogenous factors such as growth regulators and auxins play a critical role in root branching and differentiation (Malamy, 2005). The phytohormone auxin is considered to be one of the main growth regulator that triggers the lateral root formation (Bainbridge et al., 2008; Nibau et al., 2008). To monitor auxin activity in response to both biotic and abiotic factors, a *DR5* auxin-inducible promoter (Ulmasov et al., 1995; Chen et al., 2013) fused either to a *GUS* or a *GFP* reporter gene is used. It is also shown that microbes both pathogens and benign bacteria modulate root growth and differentiation (López-Bucio et al., 2006; Zolobowska and Van Gijsegem, 2006). Recently, it was shown that few beneficial microbes such as *Pseudomonas* sp. induce root developmental changes via secretion of diffused compounds (Zamioudis et al., 2013). It is argued that root-derived chemicals mediate belowground microbiome, but it is tempting to speculate that both biotic and abiotic factors may temporally change root-derived chemical synthesis and secretion (Badri and Vivanco, 2009).

Many biotic and abiotic stress regimes cause defensive responses in the affected plants. These responses are categorized based on the directness of their approach to alleviate the stressor. Direct defenses repel and kill enemies through the secretion of toxins, whereas indirect defenses, including the release of plant-derived chemicals, deter enemies by increasing predation pressure on an attacking herbivore (Kessler and Baldwin, 2001; Baldwin et al., 2002). However, most plants only increase production of the chemicals used in these defensive strategies when they are actually under attack. Documented in interspecies and intra-plant (within a single organism) systems, plant-derived chemicals including VOCs change plant transcriptional patterns of defense-related genes and can increase production of growth regulators related to defending against a certain stressor (Heil and Kost, 2006). Previous studies have investigated the complex chemical conduits active in the interconnected role between aboveground and belowground signaling of plants (Bezemer and van Dam, 2005). Belowground organisms can induce aboveground defense responses and vice versa. Exposure to damaging belowground organisms, such as insects, nematodes, root pathogens, and mycorrhizal fungi, impact the aboveground defense responses and induce indirect defenses that attract carnivores or enhance the effectiveness with which those carnivores consume the attacking herbivores. Similarly, above-ground herbivory can influence the concentration of defense-related compounds in belowground root structures (van Dam, 2009). It is clearly shown that plants can sense microbial neighbors and modify the root-derived chemicals (Badri and Vivanco, 2009). It is shown that *Arabidopsis* and *Medicago* each treated with a pathogen (*Pseudomonas syringae* DC3000) and a benign (*Sinorhizobium meliloti*) microbe trigger secretions of different proteins, indicating plants use different chemicals to signal different neighbors (De-la-Peña et al., 2008). It is appropriate to speculate that plants may have similar kind of machinery to sense the neighboring plants. On the similar lines, *Arabidopsis* plant grown in larger monocultures produce more defense metabolites (glucosinolates) compared to smaller monocultures (Wentzell and Kliebenstein, 2008).

In the present study, we speculated that plants sharing the space with a mechanically injured neighbor may show differences in root plasticity. We also questioned how the recipient community perceives damaged-derived chemical signals and evaluated its impact on root growth and root–microbe interactions. The current study relies on measurements of root growth rate and fluorescence assays using the β-glucuronidase (GUS) reporter gene in two transgenic reporter lines of *Arabidopsis thaliana* (*ALMT1::GUS* and *DR5::GUS*). These transgenic reporter lines offer insight on two belowground, induced-defense mechanisms observed in unwounded plants exposed in close proximity to injured neighbors. The triggered defense responses include the upregulation of the *ALMT1* gene and an auxin-responsive *DR5* gene, and accelerated lateral and primary root growth. We report an unusual shoot-to-root interplant communication leading to altered belowground root responses and benign biotic associations.

## Materials and Methods

### Plant Growth Conditions

Seeds of wild-type *Arabidopsis thaliana* ecotype Columbia (Col-0) were obtained from the *Arabidopsis* Biological Resource Center (ABRC) and surface sterilized using 50% sodium hypochlorite for 1 min and then thrice washed with sterile water. *ALMT1*::GUS and *DR5*::GUS transgenic lines were obtained from Hiroyuki Koyama (Gifu University, Japan) and Wendy Peer (University of Maryland). The seeds were cultured on Murashige and Skoog (MS) (Murashige and Skoog, 1962) solid agar with 3% sucrose in petri dishes and were incubated at 21 ± 2°C with 12/12 h of light and dark photoperiod and illuminated with cool fluorescent light with an intensity of 120 μEm^-2^s^-1^. At 8 days post-germination, seedlings were individually transferred to either undivided (on which two seedlings were positioned either 2 or 4 cm apart) or partition petri plates (with one seedling on each side of the partition).

For root colonization, *ALMT1*::GUS, and *DR5*::GUS assays, 12-days-old seedlings were transferred from solid MS media to 6-well culture plates (Fisher Scientific) containing 2.5 mL of 0.5x MS liquid medium with 0.05 mM MES and 3% sucrose. Each 6-well plate contained two seedlings, placed in corner wells opposite and diagonal from one another to maximize distance apart. Plants were grown for 12 days with constant shaking at 90 rpm.

### Mechanical Wounding

Sterilized, room-temperature forceps created 4 distinct punctures to the lamina of 2 of the first true leaves on each “donor” *Arabidopsis* plant. In both the Col-0, and *ALMT1*::GUS assay experiments, one seedling in each petri plate was designated the “Donor” community and was mechanically wounded, while its adjacent seedling was left untouched. Mechanical wounding occurred on the same day as seedling transfer. Non-invasive (non-puncturing) contact of the forceps on the seedlings established control trials in which neither seedling was wounded. Primary root growth rate was measured and calculated as μm h^-1^ over 8 days post-wounding.

### ALMT1::GUS Assay and Analysis

Partition plates sealed with Parafilm M^®^ film (Bemis) divide agar but allow for shared airspace. The plates used for the *ALMT1*::GUS assay were half-filled with a solid MS agar with 3% sucrose, while the other halves of the plates were filled with MS agar with 10 μM AlCl_3_ (Sigma-Aldrich). Two seedlings, 8-days post-germination, were transferred to the plates and allowed to grow per the earlier growth conditions. Mechanical wounding of the randomly selected “donor” seedling (that which “donates” VOCs) occurred on the same day as transfer. Eight days after transfer, the unwounded seedling in each plate was processed per the published description of the β-Glucuronidase Reporter Gene Staining assay (Sigma-Aldrich) and stored at 4°C in a 4% formaldehyde solution until microscopy on an AxioCam color dissecting microscope.

### *Bacillus subtilis* UD1022 Biofilm Formation

*Bacillus subtilis* UD1022 was streaked from a -80°C glycerol stock onto a plate of low-salt Luria Bertani (LB) medium (10 g L^-1^ Tryptone, 5 g L^-1^ yeast extract, 5 g L^-1^ NaCl, pH = 7.0) and grown for 24 h at 28°C with shaking at 180 RPM. A subculture was started in 200 mL of LB liquid culture from the previous streak. After shaking for 24 h at 28°C, the subculture was diluted 1:1000 and incubated further at 28°C. When the subculture OD_600_ reached 0.6–1.0, 10 μL of inoculum (OD_600_ = 0.007 of UD1022) were added to the existing 0.5X MS liquid medium in the wells of the “recipient” *A. thaliana* plants.

### Microscopy

Adherent UD1022 cells and biofilm on root surface were imaged using laser scanning confocal microscopy. After 24 h shaking at 90 rpm and 6 h stationary at 21 ± 2°C under the photoperiod described for growth conditions, UD1022-inoculated plants were removed from media and roots were sliced from aboveground plant body. Root samples were then placed in sterile 1-mL tubes (Eppendorf), rinsed once with Phosphate Buffer (2.5 mM), and then suspended in 1-mL of buffer. Histological staining relied on 1:1000 concentration STYO^®^13 (Invitrogen, Molecular Probes, Eugene, OR, USA) and 1:500 concentration Calcofluor (Sigma-Aldrich), which were left in contact with the roots for 20 ± 3 min and then rinsed once with sterile water. Images were captured with a 25X C-Apochromat objective on a Zeiss LSM 710. Spectral data was collected on the 710 spectral detector. Collected spectral data was used in online fingerprinting and images were post-processed channel unmixed resulting in blue (calcofluor), green (SYTO^®^13 in UD1022 biofilm) and red (auto-fluorescence) layers. Limited amounts of SYTO^®^13 are suspected to have penetrated root vascular tissue and cause increased green fluorescence outside of the UD1022 biofilm.

### DR5::GUS Assay and Analysis

Two seedlings, 15-days post-germination, were transferred to partition plates with a solid MS agar with 3% sucrose (Sigma-Aldrich) and allowed to grow per the earlier growth conditions. Mechanical wounding of the randomly selected “donor” seedling occurred on the same day as transfer. Five days after transfer, the unwounded seedling in each plate was processed per the published description of the β-Glucuronidase Reporter Gene Staining assay and stored at 4°C in a 4% formaldehyde solution until microscopy on an AxioCam color dissecting microscope.

### Statistical Analysis

The data were analyzed by a one-way analysis of variance (ANOVA) using JMP^®^ Pro, Version 11 (SAS Institute, Inc., Cary, NC, USA 1989–2007). When necessary to compare two means, Student’s two-tailed *t*-test were also generated using JMP^®^ Pro, Version 11.

## Results

Mechanical wounding of *A. thaliana* plant facilitated the release of airborne VOCs that induced an elaborate series of defense-mechanisms in the neighboring seedlings. The VOCs upregulated the root-specific malate transporter (*ALMT1*) gene, increasing recruitment of a beneficial bacterium, and the *DR5* auxin promoter, which accelerated the root growth.

### Airborne Volatile Organic Compounds (VOCs) from Mechanical Wounding Accelerate Root Growth

**FIGURE 1:**
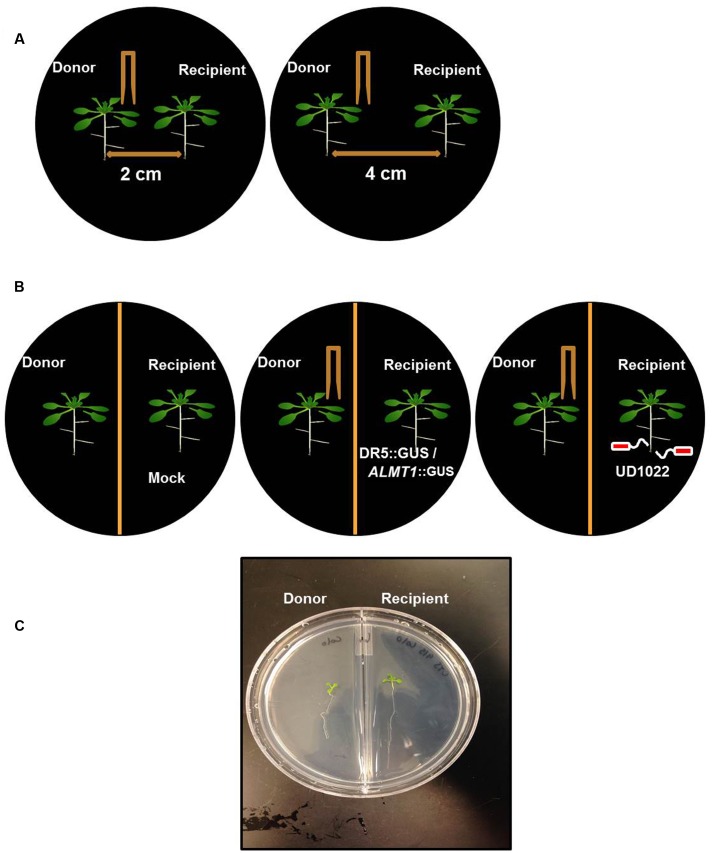
**Schematic of *Arabidopsis thaliana* seedlings of donor and recipient communities in different experiments.** **(A)** Twelve day old, two uniform *Arabidopsis* seedlings were transferred to a petri plate with 2 cm and 4 cm apart from each other on petri plates. Donor seedling was wounded to induce VOC release and growth of the seedling was recorded after 8 days of wounding. **(B)** Similarly, *Arabidopsis* seedlings were transferred on the either side of partition petri plates. The donor plants were Col-0 and wounded and recipient’s plants were DR5::GUS or ALMT1::GUS or Col-0 rhizo-inoculated with UD1022. **(C)** An image of the recipient and the donor community of *Arabidopsis* in a partition plate.

**FIGURE 2:**
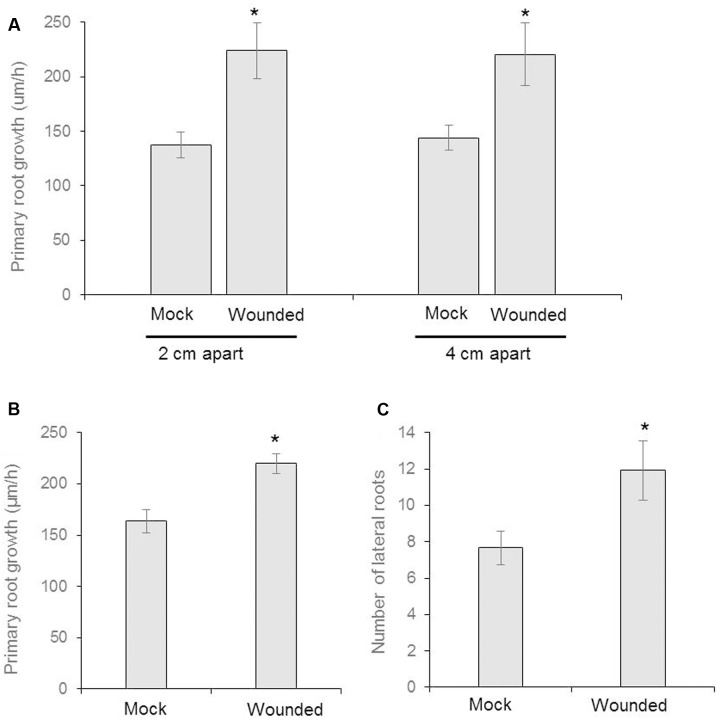
**Seeds of *Arabidopsis* (Col-0) were germinated and two uniform seedlings of 12 days old were transferred to the diffused plate or partitioned plate and placed 2 cm or 4 cm apart each other (as shown in schematic **Figure 1A**).** At the same time, the donor plants were wounded mechanically and incubated for 8 days. **(A)** The growth rate of primary root of recipient plant in diffused plate and presented as cm/h (*n* = 6). **(B)** The measurement of growth rate of recipient plant in partitioned petri plate (*n* = 12). **(C)** Number of lateral roots in recipient plants (*n* = 12). Asterisks denote significant differences as analyzed by Student’s *t*-test. ^∗^*P* ≤ 0.05, Student’s two-tailed *t*-test. Error bar standard error mean.

We designed a prototype to test if wounding neighboring plants changes belowground phenotype in the neighboring communities (**Figures 1A,C**). The Recipient (the unwounded seedling) and Donor (the seedling that is wounded and releases VOCs) seedlings were positioned either at 4 or 2 cm apart from each other, in an effort to identify any changes in response that may be due to weakened potency of the VOC signal over greater distance between plants. We used partition- and no-partition- prototypes to check if the Donor community releases both VOCs and root exudates to trigger change in phenotype in the Recipient communities (**Figure 1B**). We speculated that the no other signals besides VOCs could be exchanged in the partition plates, hence VOCs may play a critical role in signaling between injured donors and recipient communities. Mechanical wounding of the Donor seedling was followed by the observation that, at 4 cm apart, the primary lateral roots of Recipients next to wounded Donors grew significantly faster at 224 ± 63 μm h^-1^ than the 137 ± 29 μm h^-1^ of the seedlings next to unwounded Donors. There was, however, no significant difference in the growth rate of primary lateral root (“PR”) between the 2 and 4 cm spaced trials (**Figure 2**). This result necessitates further investigation into the potency of the VOC signal over larger distance. The number of lateral root extensions (“LR”) on the Recipient seedlings was also counted, with Recipient communities next to wounded Donors showing an average of 4.25 more lateral roots than Recipients with unwounded Donors (**Figure 2**).

Partition plates were used to assess whether the noted VOC signal functioned as a diffusion signal and would still affect the Recipient community without sharing the same medium (**Figure 1B**). Recipient plants next to wounded Donors exhibited accelerated root growth consistent with that observed in the undivided petri plates: the mean primary root growth rate was 220 ± 9 μm h^-1^ compared to the control growth rate of 164 ± 11 μm h^-1^ (**Figure 2**). A strong causational pattern is established here between exposure to the VOCs elicited by mechanical wounding and acceleration of growth in PR and LR, suggesting a commensalistic relationship in which wounded plants signal potentially vulnerable neighboring plants of the same species to mitigate damage by increasing biomass.

### Auxin Response Upregulated in Presence of VOCs

**FIGURE 3:**
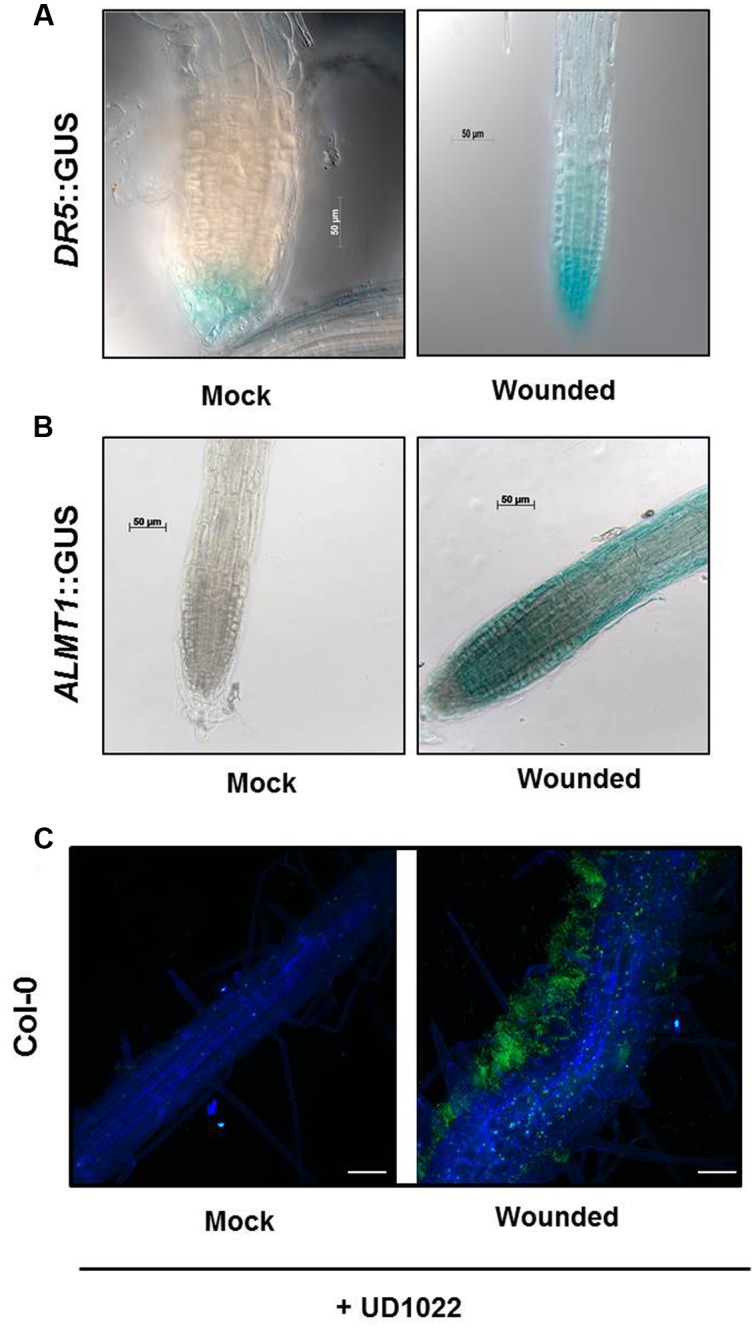
**Seeds of *Arabidopsis* (Col-0) or *DR5*::GUS or *ALMT1*::GUS were germinated and two uniform seedlings of 12 days old were transferred to the partitioned petri plate (as shown in schematic **Figures 1B,C**).** The Donor plants were wounded 2 days after transfer and allowed to sit for 24 h prior to staining. **(A)** GUS staining of recipients *DR5*::GUS seedlings was performed 24 h post-treatment. Scale bars = 50 mm. **(B)** GUS staining of recipients *ALMT1*::GUS seedlings was performed 24 h post-treatment. Scale bars = 50 mm. **(C)** Seeds of *Arabidopsis* Col-0 were germinated and two uniform seedlings of 12 days old were transferred to the 6-well culture plates. The Donor plants were wounded, and recipients plants were rhizo-inoculated with UD1022 (OD600 = 0.001) for 24 h. The green fluorescence in the panels shows UD1022. Bars = 50 mm.

Both Donor and Recipient groups in the wounded treatment exhibited longer PR and a greater number of LR than the control Donor and Recipient groups, suggesting that the VOCs released by mechanical wounding may upregulate the auxin response that results in increased accumulation of auxin in the apical meristem of primary roots in both the Recipient and Donor plants. To confirm the involvement of auxin interplay in the VOC triggered Recipient communities, we used an auxin reporter *DR5*::GUS line. Recipient communities described in **Figure 1** adjacent to the wounded/unwounded Donor communities were replaced by the *DR5*::GUS lines. 24 h post-wounding, *DR5*::GUS expression of the roots of the Recipients next to wounded Donors exhibited deeper blue coloration, suggesting greater auxin accumulation than in the roots of the control (**Figure 3A**). Blue staining was concentrated in gradients toward root apex of the primary and lateral root extensions. The significant difference in the magnitude of gradient in the GUS staining suggests that a signaling cascade for the *DR5* auxin reporter is triggered by the VOCs documented in this study; the *DR5* upregulation is consistent with the earlier observation that primary root and lateral root extensions were longer and more numerous in our earlier experiments.

### VOCs Upregulate Malate Transporter in the Recipient Communities

Previously, we have shown that root specific malate transporters (such as *ALMT1*) play a vital role in shaping root microbiome in plants infected with an aboveground pathogen (Rudrappa et al., 2008). We also show that the wound-induced VOCs change root phenotype in the Recipient communities, which may involve auxin interplay. Here we argued that the wound-induced VOCs may also modulate *ALMT1* expressions in the neighboring communities. To this end, the neighboring plants exposed to wounded and unwounded Donors were replaced by *ALMT1*::GUS expressing reporter lines. *ALMT1*::GUS expressing reporter lines exposed to wounded/unwounded Donors were harvested post 24 h of exposure. *ALMT1*::GUS expressing reporter lines were stained for GUS activity per the published protocol (Sigma-Aldrich, Co.; Rudrappa et al., 2008). Recipients next to unwounded Donors served as controls. *ALMT1*::GUS expression of the roots of the Recipients next to wounded Donors stained deeper blue than the roots of the control, which exhibited no GUS fluorescence (**Figure 3B**). GUS expression was concentrated in lateral root extensions and the apical meristem of the primary root, suggesting activation of malate transporter by wounded-induced VOCs.

The intensity of the GUS staining in the Recipient plants next to wounded Donors strongly indicates the existence of a signal transduction pathway that upregulates the *ALMT1* in the presence of the VOC’s elicited by mechanical wounding.

### Recipient Plants Exhibited Increased Association with Benign *Bacillus subtilis* UD1022 in Presence of Wound-induced VOCs

Having shown that wound-induced VOCs trigger malate transporter expression in the Recipient communities, we tested whether the increased malate transporter expression triggered more root colonization by benign bacterium *B. subtilis* UD1022 in the Recipient communities. Recipient communities exposed to wounded/unwounded plants were subjected to UD1022 inoculum (OD_600_ = 0.007 of UD1022). Post 24 h of exposure to the wounded/unwounded plants’ VOCs, Recipient communities were checked for *B. subtilis* colonization and biofilm formation using confocal microscopy. Confocal imaging of Recipient plant roots exposed to the wounded Donors revealed significantly more UD1022 biofilm development than existent on control roots (exposed to the unwounded Donors) inoculated with identical levels of UD1022 (**Figure 3C**). This result suggests that malate upregulation induced by wounded Donors may also associate more with the beneficial bacterium UD1022.

## Discussion

The impact of aboveground to belowground signaling in intra-plant communications is a field that has recently gained a lot of attention. Both biotic and abiotic stress trigger mobile signals between the aerial and root parts of a plant (Pangesti et al., 2013) Various studies have shown a two-way communication conduit in plants, wherein plants exposed to aerial pathogens and herbivory alter root phenotype and roots exposed to both biotic and abiotic stress change aboveground physiology in plants (Pangesti et al., 2013). Similarly, few lines of studies have shown that interplant communications changing aboveground physiology in plants exposed to both biotic and abiotic stress agents (Heil and Bueno, 2007). Earlier work explored the concept of “talking trees,” introducing the prey-parasitoid concept triggered by release of VOCs from the stressed plants (De Moraes et al., 2001). The majority of work related to interplant communication relates to VOC-inducible defense responses in plants (Paré and Tumlinson, 1996). It has been demonstrated that VOCs can attract predatory parasitoids, thus mitigating the threat of attacking herbivores (Thaler, 1999; De Moraes et al., 2001; Kessler and Baldwin, 2001; Karban, 2011). In contrast, it has also been shown that VOCs can help herbivores locate hosts, leading to plant damage (Horiuchi et al., 2003). Most interestingly, VOCs can be used by neighboring, yet-undamaged plants in proximity to damaged plants to adjust their defensive phenotypes (Heil and Bueno, 2007). So far, leaf-derived VOCs in interplant communication have resulted in changes to aboveground physiology. Similarly, involvement of leaf-derived VOCs in intraplant communication has shown microbiome shifts and plant defense response (Song et al., 2016). There exists a gap in our knowledge of how VOCs may modulate interplant communication by changing belowground plasticity and root–microbe interactions. The current study shows that VOCs derived from a damaged plant change root plasticity and root–microbe interaction in the neighboring, yet undamaged plants.

Wounding response at the intraplant level is very well-characterized. It has been shown that wounded plants trigger both local and systemic responses at the intra-plant level (León et al., 2001). It has also been shown that various growth regulators play a part in wound signaling in plants (León et al., 2001). Agents such as oligosaccharides (OGAs), ethylene, jasmonic acid (JA), and abscisic acid (ABA) play a critical role in those local and systemic responses during wound signaling; the signal peptide systemin has also been demonstrated to influence defensive responses in a wounded plant system (León et al., 2001). Systemin is an 18-amino acid peptide generated from a larger protein precursor called prosystemin (McGurl and Pearce, 1992) and is known to modulate growth regulators in wounded plants. It was reported that wounding in plants causes an increase in ethylene concentration, leading to altered defense responses in plants (Liu et al., 1993; Bouquin et al., 1997). In contrast, wounded tobacco plants show decreased auxin responses (a drop in the endogenous levels of indole acetic acid) (Thornburg and Li, 1991). It has been proposed that the recovery of the initial levels of active auxins serves as a mechanism to limit the duration of the response to wounding (Rojo et al., 1998). Our results showed a contrasting phenotype in the undamaged plants exposed to the wounded neighbors. Plants exposed to the wounded neighbors showed an increase in root growth compared to plants exposed to undamaged neighbors. The shift in plasticity at the interplant level showed that plants respond to aboveground VOCs and alter belowground phenotype. Though auxins are reported to show an inverse relationship with wound response at the intraplant level, our results showed that auxin may play a different role in the interplant signaling response. The *DR5* gene marked with the GUS reporter gene in this study is a synthetic auxin-responsive promoter that indicates high auxin accumulation (Chen et al., 2013). In our study, the increase in *DR5*::GUS staining evident in the root cap and apical meristem of Recipient seedlings next to the wounded Donors indicates that the VOCs initiate the signal transduction responsible for the upregulation of *DR5*. This histochemical evidence compliments the accelerated growth rate (a sign of augmented auxin accumulation) seen in the Recipients in proximity to wounded Donors. The increase in the auxin-responsive promoter *DR5* in roots of neighboring plants exposed to the damaged neighbors suggests that auxin activity operates differently under interplant wound signaling compared to intraplant wound signaling.

Our work adds a layer of complexity to the previously documented above- and belowground interactions involving VOCs and microbiome interactions. Recipient plants exposed to damaged neighbors showed increased *ALMT1* expression followed by increased colonization by UD1022. The matrix of cells (see green in **Figure 3C**) surrounding only the Recipient root next to a wounded neighbor illustrates the increased recruitment of *B. subtilis* associated with the upregulation of *ALMT1*. Previous research has shown that this strain is an effective plant growth-promoting rhizobacteria and reduces foliar entry of deleterious pathogens (Kumar et al., 2012). It is also shown that colonization by beneficial microbes on the root surface increases plant growth promotion and bioprotection activity in plants (Lakshmanan et al., 2014; Allard-Massicotte et al., 2016). The literature suggests that the impact of beneficial microbe-derived volatiles on plants may play a critical role in inducing plant growth promotion and biocontrol activity (Ryu et al., 2003; Rudrappa et al., 2010; Kanchiswamy et al., 2015; Zamioudis et al., 2015). Conversely, there is a gap in our understanding in terms of plant sentinels that may trigger association of benign microbial association with plants (Lakshmanan et al., 2014). We have evidence of how plants manipulate belowground microbiome (Lakshmanan, 2015; Wagner et al., 2016), but we still lack data of plant-derived factors that may modulate the microbiome diversity. There is also evidence which shows that association of beneficial microbes in plants is not a straight-forward process and involves suppression of defense response in plants by benign microbes (Lakshmanan et al., 2012). It would be interesting to see if suppression of defense response by benign microbes also exists in studies involving plant communities. Our work showed that plants may relay a stress-induced sentinel which attracts belowground benign microbes in the neighboring yet undamaged plants. At this juncture we do not fully understand how this association may inflict plant growth promotion phenotype in the undamaged neighboring plants.

In this study, we report that aboveground mechanical wounding elicits substantial belowground changes in plant phenotypic and genotypic characteristics. Previous literature has shown the defense-catalyzing capabilities of VOCs on intraplant and interspecific systems (Holopainen and Gershenzon, 2010). Likewise, the effect of mechanical wounding has been noted to induce upregulation of genes in local, undamaged seedlings (Heil and Kost, 2006). The novelty of the current study lies therein the observation, that those priming VOCs reflect altruistic evolutionary developments, as the plant is purposefully designed to warn its neighbors in light of its own damage. The upregulation of malate transporter and auxin responsive genes in neighboring plants exposed to wounded neighbors suggests that *A. thaliana* evolved to anticipate abiotic and biotic stress and survived most when root systems matured at a faster rate, allowing for adequate nutrient and moisture uptake even in potentially contaminated soil.

## Conclusion

Our hypothesis that the same defense response elicited by VOCs in intraplant and interspecific systems would be induced between neighboring, but anatomically separate plants was correct. We identified the benefits of the *ALMT1* malate transporter in increasing biofilm development and the *DR5* auxin reporter in accelerating root growth. These findings contribute to a growing body of research on root–microbe interactions and expand the agricultural applications of VOCs as factors for pathogen protection and plant growth promotion. Our study demonstrates that the volatiles released by damaged plants elicit belowground changes related to root plasticity and root–microbe interactions in neighboring plants under controlled conditions. Many questions still remain regarding the capabilities of this specific aboveground-belowground VOC relationship: how concentrated does the VOC signal have to be to effectively upregulate auxin-responsive and malate transporter genes under natural conditions? What is the specific chemical composition of the VOC signal at play in this interplant interaction? What other genotypic transductions do these VOCs cause, aside from the two genes of interest reported in the present study? Do VOCs derived from wounded plants play a role in belowground interspecies signaling? The answers to these inquiries ultimately march closer to a new breed of organic crop primers that secure more robust, disease-free crop yields without relying on unsustainable industrial fertilizers.

## Author Contributions

CS conducted all the experiments described in the manuscript. VL and CS analyzed the data. CS performed the statistics on the root growth analysis. CS and HB drafted the manuscript. HB conceived the study and CS participated in its design and coordination. All authors read and approved the final manuscript.

## Conflict of Interest Statement

The authors declare that the research was conducted in the absence of any commercial or financial relationships that could be construed as a potential conflict of interest.
